# CNGCs break through—A rice cyclic nucleotide-gated channel paves the way for pollen tube growth

**DOI:** 10.1371/journal.pgen.1007066

**Published:** 2017-11-30

**Authors:** Wolfgang Moeder, Keiko Yoshioka

**Affiliations:** 1 Department of Cell and Systems Biology, University of Toronto, Toronto, Ontario, Canada; 2 Center for the Analysis of Genome Evolution and Function (CAGEF), University of Toronto, Toronto, Ontario, Canada; University of Arizona, UNITED STATES

## Rice cyclic nucleotide-gated channel 13 is a novel player in pollen tube-pistil signaling

Lower plants generate mobile sperm cells that must reach their female counterparts by swimming. This requirement for water is a disadvantage for these plants as compared with angiosperms, for which the dry pollen attaches to a stigma and becomes hydrated, enabling the emerging pollen tube to grow in the protected environment of the pistil. After adhesion, hydration, and germination of the pollen at the stigmatic papilla cells, the pollen tube enters the stigma and grows in the intercellular space between papilla cells towards the style and transmission tract (TT). The TT contains a nutrient-rich extracellular matrix (ECM) and guides the pollen tube to the ovary. After penetrating the septum, the pollen tube grows through the funiculus and then enters the ovule though the micropyle to deliver the two nonflagellate sperm cells to the two female gametes, leading to double fertilization, a prerequisite to seed formation [1]. The 2017 study by Xu et al. [2] reveals, for the first time, the importance of a Ca^2+^ signal generated by rice cyclic nucleotide-gated channel 13 (*OsCNGC13*) in the pistil to induce programmed cell death (PCD), which facilitates proper pollen tube growth. Furthermore, they showed this step significantly affects the yield of rice grains.

**Fig 1:**
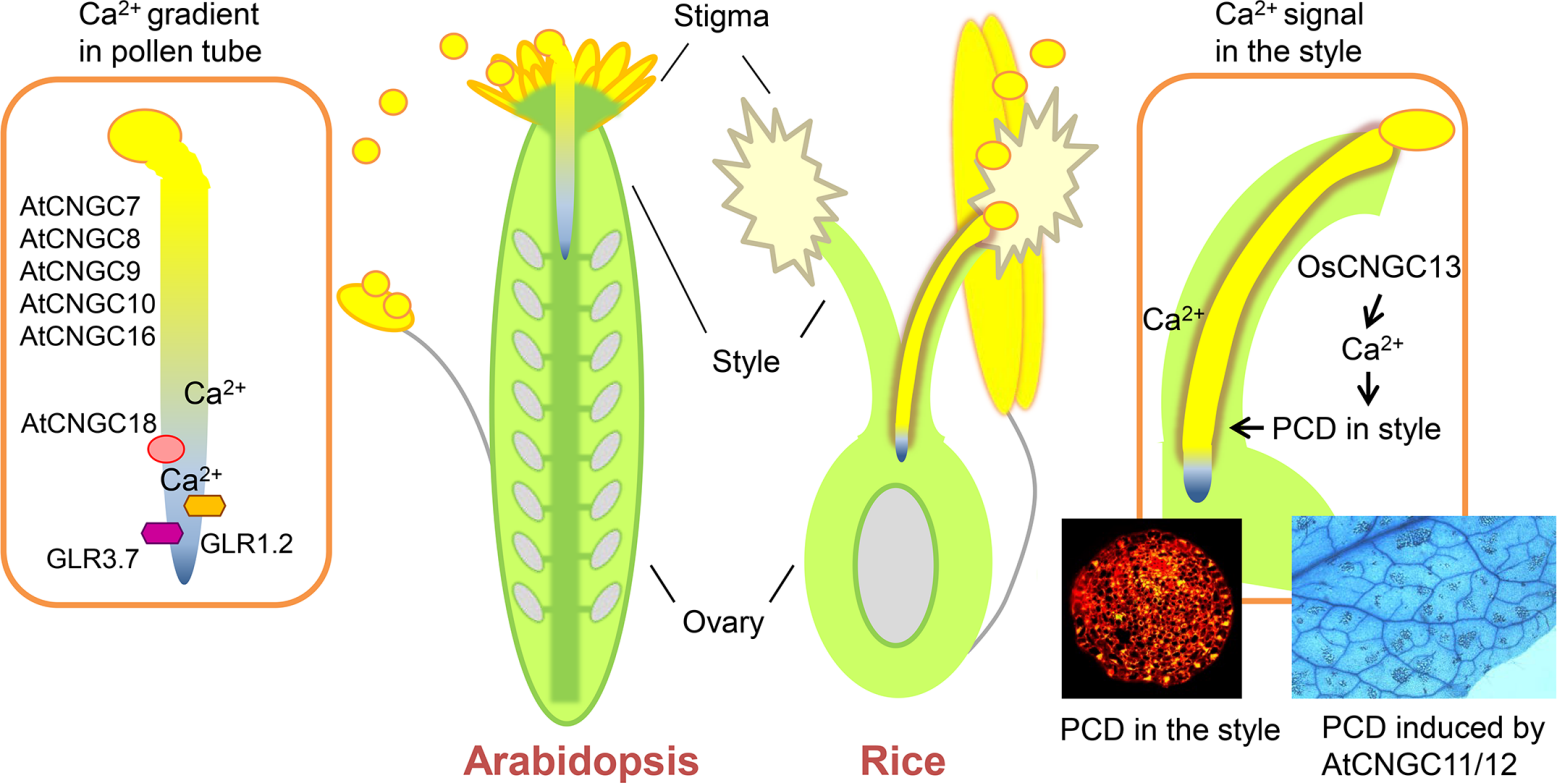
Ca^2+^ channels in pollen tubes and pistils in *Arabidopsis* and rice. The Ca^2+^ gradient in pollen tubes is created by members of the CNGC and GLR families (left). Xu et al. [2] reveal a role of *OsCNGC13* in the TT of the rice style, where a CNGC-mediated Ca^2+^ signal is required to induce cell death, which is necessary for the pollen tube to grow through the TT to reach the ovary (right). A role for CNGCs in PCD formation had been previously shown for *AtCNGC11/12* [21]. CNGC, cyclic nucleotide-gated channel; GLR, glutamate receptor-like; PCD, programmed cell death; TT, transmission tract.

The events during pollen grain–stigma interaction and pollen tube reception are relatively well studied [1, 3], while much less is known about the growth of the pollen tube through the style and TT tissue—particularly the signaling between the pollen tube and the pistil tissue(s). Intracellular signaling in the pollen tube during pollen tube growth has been studied extensively [4]. The role of Ca2+ is well established: in the pollen tube, a Ca2+ gradient is essential for pollen tube guidance [5]. External Ca2+ from the pistil must be taken up by the pollen tube and is required for its growth. Several potential Ca2+ channels that are expressed in the pollen tube have been identified in *Arabidopsis*. These include two glutamate receptor-like (GLR) channels (*GLR1*.*2* and *GLR3*.*7*) [6] and six cyclic nucleotide-gated channels (CNGCs; *CNGC7*, *8*, *9*, *10*, *16*, and *18*), of which *CNGC18* has been shown to be a Ca2+-conducting channel that is essential for tip growth in pollen tubes [7] and pollen tube guidance (Fig 1) [5].

Pollen tubes can grow *in vitro*; however, for the pollen tube to make its way through the style to the ovary, some communication with the sporophyte is necessary. But so far, few signaling components have been identified that drive the interaction between the TT and the pollen tube. On the pollen tube side, two membrane-localized leucine rich repeat (LRR) receptor kinases, *LePRK1* and *2*, have been identified that may interact with different ligands in different pistil tissues [8]. Other examples include the pollen tube-localized GLRs, *GLR1*.*2* and *GLR3*.*7*. They are activated by D-serine, which is produced in the TT by the serine racemase *SR1*, which converts L-serine to its biologically active enantiomer [6]. The C2H2/C2HC zinc finger transcription factor *No Transmitting Tract* (*NTT*) has been identified as a key regulator of the ECM tissue; pollen tubes will terminate prematurely in *ntt* mutants [9]. Other factors in the TT that affect pollen tube growth are three *HECATE* transcription factors and the auxin response factors *ARF6* and *8* [10].

Xu et al. show that the *semi-seed-setting rate1*-*Dominant* (*sss1-D*) rice mutant displays a low seed yield phenotype, caused by premature termination of pollen tube growth, which was connected to delayed PCD in the style. The causal mutation was mapped to *OsCNGC13*. An inversion of a DNA segment caused a truncation of this gene, resulting in the OsCNGC13-D protein with only 440 amino acids with five transmembrane domains but lacking the pore-forming region and the cytosolic C-terminal domain, which contains important regulatory domains such as cyclic nucleotide-binding domain (CNBD) and calmodulin binding domain(s) (CaMDB) [11]. The truncated protein does not act as a dominant negative because it did not bind to wild-type (WT) OsCNGC13 (although this aspect may need to be confirmed by additional experiments), but rather the expression of the truncated transcript seems to lead to a down-regulation of WT *OsCNGC13*, potentially through an RNA interference (RNAi) mechanism.

Thus, the dominant nature of *sss1-D* can be explained by haploinsufficiency of WT *OsCNGC13*. The levels of WT transcript are decreased in heterozygous plants as well as in *OsCNGC13-D*–overexpressing plants, while knocking out *OsCNGC13* phenocopies the *sss1-D* mutant phenotype.

Increased expression of *OsCNGC13* was detected in WT pistils 30 minutes after pollination. Patch-clamp whole cell recordings in HEK293 cells detected inward Ca2+ but not K+ currents. Furthermore, an increase in [Ca2+]cyt at the bottom of the style after pollination was shown, which was absent in *sss1-D* plants. Taken together, they concluded that a Ca2+ signal in the pistil mediated by *OsONGC13* is required for normal pollen tube growth.

## CNGC-mediated PCD in pollen tube growth and immunity

What makes this finding relevant for the broader CNGC research field is the connection between Ca2+ influx and PCD. At 30 minutes after pollination, TUNEL staining revealed small patches of dying cells in the style that widen the intercellular space, allowing the pollen tube to grow through the ECM (Fig 1). Prior to this study, several CNGCs had been connected to PCD and plant immunity, particularly *AtCNGC11* and *12* (Fig 1) of group I and *AtCNGC2* and *4* of group IVb [12]. *OsCNGC13* is the rice ortholog of *AtCNGC19*, which belongs to group IVa. This is the first report to connect a group IVa channel to PCD.

So far, the role of *AtCNGC19* has not been well established. It has been connected to NaCl stress responses [13], and gene expression studies show that it is up-regulated after pathogen treatment(s). Furthermore, *AtCNGC19* KO lines also exhibit enhanced susceptibility against the necrotrophic pathogen *Botrytis cinerea* [12]. Interestingly, *OsCNGC13* is also induced by a pathogen, *Pseudomonas fuscovaginae* [14], suggesting a role in immunity as well. This may indicate that the biological function of IVa CNGCs is conserved among different plant species, thus it will be interesting to know whether *AtCNGC19* and *20* are also involved in PCD and/or pollen tube growth.

## What can we learn regarding function and regulation of CNGCs?

Group IV CNGCs are the most divergent group of CNGCs, with two subclasses, IVa and IVb. The closest paralog to *AtCNGC19*—which is the orthlog of *OsCNGC13*—and sole other IVa group member is *AtCNGC20*, which is the ortholog of *OsCNGC12* [14]. Loss-of-function mutants of *AtCNGC2* or *4*, which make up group IVb, show a clear morphological phenotype and display constitutive cell death [15]. The barley lesion mimic phenotype of the *nec1* mutant is also caused by a mutation of the barley ortholog of *AtCNGC4*, indicating functional conservation in monocots [16]. Interestingly, *AtCNGC2* has been connected to reduced fertility. Pollen tubes in *cngc2* mutant pistils exhibited large rates of premature pollen tube termination prior to reaching the ovules, which was even more pronounced under increased Ca2+ concentrations [17].

The group IVa CNGCs differ from other CNGCs in two aspects. First, they possess a substantially larger N-terminal cytosolic domain (172–204 amino acids versus 40–100 for other CNGCs). Interestingly, a CaMBD is predicted for the AtCNGC19/OsCNGC13 N- termini (http://calcium.uhnres.utoronto.ca/ctdb/pub_pages/search/index.htm), although no CaM binding has been shown yet. Since they also possess a C-terminal isoleucine glutamine (IQ) domain (a type of CaMBD) [18], they may have a similarly complex regulation by calmodulin, as previously shown for *AtCNGC12* [11].

Second, class IVa CNGCs have 10 additional amino acids in their CNBD between the phosphate binding cassette (PBC) and the hinge motifs, suggesting that this group is regulated differently than the other CNGCs [19].

In summary, the 2017 study by Xu et al. [2] connects [Ca2+]cyt accumulation, ECM composition, and PCD in the style after pollination, paving the way for further research into how signal transduction in the style allows the pollen tube to penetrate the style and TT. Furthermore, it demonstrates a function for another member of the CNGC family. Most of the family members (20 in *Arabidopsis*, 16 in rice) have not been functionally characterized, though there has been significant progress recently [20]. Future research challenges include uncovering the regulation of CNGC channel function by cyclic nucleotides (or other ligands) and the Ca2+ sensor protein, calmodulin, as well as determining whether CNGCs form homo- or hetero-tetrameric channels.
